# Massachusetts Dental Society

**Published:** 1900-05

**Authors:** 

**Affiliations:** Massachusetts Dental Society


### MASSACHUSETTS DENTAL SOCIETY.

(Continued from page 133.)

Owing to the late hour, Dr. Bogue's paper, on the "Consequences of the Extraction of Permanent Teeth," was not discussed. (For. Dr. Bogue's paper, see page 305.)

### CLINICS.

Dr. W. I. Brigham, of South Framingham, demonstrated a new cervical clamp, a gold holding attachment for mouth mirror, together with some specimens of finished work. He also gave a clinic on burnishing gold fillings.

Dr. P. W. Soule, of Monson, gave a clinic illustrating the use of soft pine wedges.

Dr. F. L. Marshall, of Boston, gave a clinic on his new method of putting in porcelain inlays in approximate cavities in front teeth, in such a manner that they are practically as durable as all gold filling. It is a combination of porcelain and gold. The gold occupies all the cavity except that in the line of vision looking at the front of the tooth. The gold is put in in such a way that it forms a box for the porcelain to set in. Dr. Marshall also gave an exhibition of staple crowns. These furnish the basis for bridge work, and have the merit of showing no gold on the face of the tooth.

Dr. Burton C. Russell, of Keene, N. H., gave a clinic on inlaid porcelain work an approximate cavities.

Waldo E. Boardman, D.M.D.,
*Editor Massachusetts Dental Society.*

